# High-pressure and high-temperature polymorphism of Na_2_CO_3_ up to 10 GPa

**DOI:** 10.1107/S2052520626004956

**Published:** 2026-06-23

**Authors:** Benedetta Chrappan Soldavini, Marco Merlini, Chiara Mangano, Davide Comboni, Wilson Crichton, Michael Hanfland

**Affiliations:** a Dipartimento di Scienze della Terra ‘A. Desio’ Università di Milano Via S. Botticelli 23 20133Milano Italy; b ESRF - European Synchrotron Radiation Facility 71 Avenue des Martyrs CS40220 38043 Grenoble Cedex 9 France; Academy of Sciences of the Czech Republic, Czechia

**Keywords:** Na_2_CO_3_, sodium carbonate, high-pressure, phase diagram, polymorphism

## Abstract

We report two new high-pressure polymorphs of sodium carbonate, ɛ and ɛ-II, stable over ∼2 GPa and 11 GPa, respectively. The ɛ polymorph exhibits a doubling of the *c* unit-cell parameter relative to the γ and β phases, crystallizes in space group *Cc*, and has a bulk modulus of 47.6 (8) GPa, comparable to that of the γ phase. Its stability is confirmed using *in situ* single-crystal and large-volume press (LVP) experiments. Phase transitions observed over a broad pressure–temperature range allow us to refine the topology of the Na_2_CO_3_ phase diagram. By integrating precise single-crystal constraints with LVP observations, we propose an updated phase diagram that shows an expanded stability field for the incommensurate γ phase, and reveals a wide stability field for the ɛ phase at conditions relevant for the Earth’s mantle. These results provide improved experimental constraints for the high-pressure behavior of sodium carbonate.

## Abstract 2

Two new high-pressure phases, ɛ and ɛ-II, observed during *in situ* experiments, complete the high-pressure phase diagram of Na_2_CO_3_ and show that both the incommensurate γ phase and the ɛ phase are stable over a wider pressure–temperature range under mantle-relevant conditions.

## 1. Introduction

Sodium carbonate (Na_2_CO_3_) draws crystallographic attention due to its complex polymorphism and modulated structure. The historical significance of this phase is due to the incommensurate structure of the room-temperature polymorph γ, whose structural solution has been complicated to finalize. Brouns & Visser (1964 ▸) reported the first description of the γ lattice underlining the necessity to use four indexes to describe the satellite reflections observed in diffraction patterns. It has been the first structure to be refined as a modulated phase using a harmonic approximation (De Wolff, 1974 ▸; Van Aalst *et al.*, 1976 ▸). Further work by Dusek *et al.* (2003 ▸) highlighted the importance of incorporating multiple modulation waves in the structure solution, distinguishing between harmonic and anharmonic descriptions.

The interest in Na_2_CO_3_ arises also from the wide number of temperature-driven phase transitions observed at atmospheric pressure, where four different polymorphs are stable (Harris & Salje, 1992 ▸):

δ (*P*2/*a*) → 130 K → γ (*C*2/*m* average) → 620 K → β (*C*2/*m*) → 763 K → α (*P*63/*mmc*).

The δ phase structure was initially described by de Pater & Helmholdt (1979 ▸) and later confirmed by Harris & Salje (1992 ▸). Its structure was then reported by Dusek *et al.* (2003 ▸), in comparison with the γ structure. While γ and δ are structurally similar, δ is a commensurate phase with lower modulation amplitudes and reduced atomic displacement parameters. The γ–β phase transition leads to a monoclinic structure, while at higher temperatures, the β–α phase transition represents a ferro­elastic phase transition (Harris & Dove, 1995 ▸; Swainson *et al.*, 1995 ▸; Harris *et al.*, 1997 ▸). More recently, Ballirano (2011 ▸) provided an in-depth structural characterization of Na_2_CO_3_ phases and their temperature-driven transitions, using *in situ* experimental techniques. The high-temperature behavior of Na_2_CO_3_ has been also presented as a model system for lattice melting (Swainson *et al.*, 1995 ▸).

De Wolff & Tuinstra (1986 ▸) provided detailed structural descriptions of the α, β and γ phases, emphasizing the role of NaO_6_ octahedral columns and their evolution under stress and modulation. They described the Na_2_CO_3_ structure as composed of sodium face-sharing octahedra columns (hosting Na1 and Na2 atoms) parallel to the hexagonal axis of the structure, interconnected by the CO_3_ groups. The space between the CO_3_ groups and the columns hosts Na3 atoms, placed in between the columns and the CO_3_ groups. Dusek *et al.* (2003 ▸) underlined the importance of the Na3 site, whose coordination environment and bond dynamics appear to drive phase transitions. In the α phase, long Na–O distances are stabilized by high thermal motion; in the β phase, shear stress brings atoms closer, with a vertical displacement of the Na columns and a reduction in symmetry; and in the γ phase, strong modulation results in variable bonding configurations. The evolving bonding behavior of this Na site possibly represents a key mechanism behind the polymorphic transitions.

From the geological perspective, the relevance of Na_2_CO_3_ becomes more pronounced at HP-HT conditions. Early studies (*e.g.* Shatskiy *et al.*, 2013 ▸) proposed a phase diagram based on energy-dispersive techniques. More robust findings from multianvil (MA) and diamond anvil cell (DAC) experiments (Gavryushkin *et al.*, 2019 ▸) based on the powder diffraction technique, suggested the stability of γ up to 12 GPa in MA experiments, with a phase transition occurring near 26 GPa. Interestingly, the γ structure remains stable up to 25 GPa in DAC experiments, suggesting discrepancies due to resolution limits in MA data, which often fails to distinguish between β and γ phases. At pressures above 12 GPa and high temperature (Gavriuskin *et al.*, 2019 ▸), the γ structure is replaced by a hexagonal phase, which is demonstrated to be the thermodynamically stable phase from those pressures to 25 GPa, where it is replaced by a higher-pressure monoclinic polymorph (Gavriuskin *et al.*, 2019 ▸).

Given the complexity of the low-pressure polymorphism, the crystallographic interest for the presence of incommensurate structure, and also the geological importance of this system, a comprehensive understanding of its phase diagram, especially in the pressure interval 0–10 GPa, is essential for both crystallographic modeling and geophysical applications.

We report an *in situ* high-pressure and high-temperature study to assess the thermo-elastic properties of Na_2_CO_3_ and its stability phase diagram. We also report two new high-pressure structures; one of these appears to be an additional stable polymorph.

## 2. Methods

Single crystals of sodium carbonate were crystallized from melting pure reagent sodium carbonate powder in a platinum crucible, reaching a temperature of 1124 K. Careful crystal selection under the microscope allowed the identification of single domains that were then used for different *in situ* single-crystal X-ray diffraction (SC-XRD) measurements (Table 1 ▸ for details).

One experiment (Run 1) was performed in a membrane Boehler–Almax design DAC with 300 µm culet diamonds and stainless-steel gasket. The cell has been loaded with a single crystal of Na_2_CO_3_ and a ruby sphere, with helium as pressure transmitting medium. SC-XRD data were collected at ID15b beamline at the European Synchrotron Radiation facility (ESRF, Grenoble, France). The beamline was equipped with a Mar555 image plate detector and data were collected with a wavelength of 0.41105 Å (Merlini & Hanfland, 2013 ▸). Pressure was increased up to 38.37 (5) GPa in small steps and for each step pressure was calibrated using ruby fluorescence (Mao *et al.*, 1986 ▸).

A second experiment (Run 2) was performed using a 600 µm culet DAC with stainless-steel gasket. In this case, a single crystal of Na_2_CO_3_ and the ruby were loaded in the cell along with silicone oil as pressure medium. Data were collected up to 8.55 (5) GPa at the ID09 beamline (ESRF, Grenoble, France). For beamline details see Merlini & Hanfland (2013 ▸). A Mar555 detector and a wavelength of ∼0.41 Å were used.

**Figure 1:**
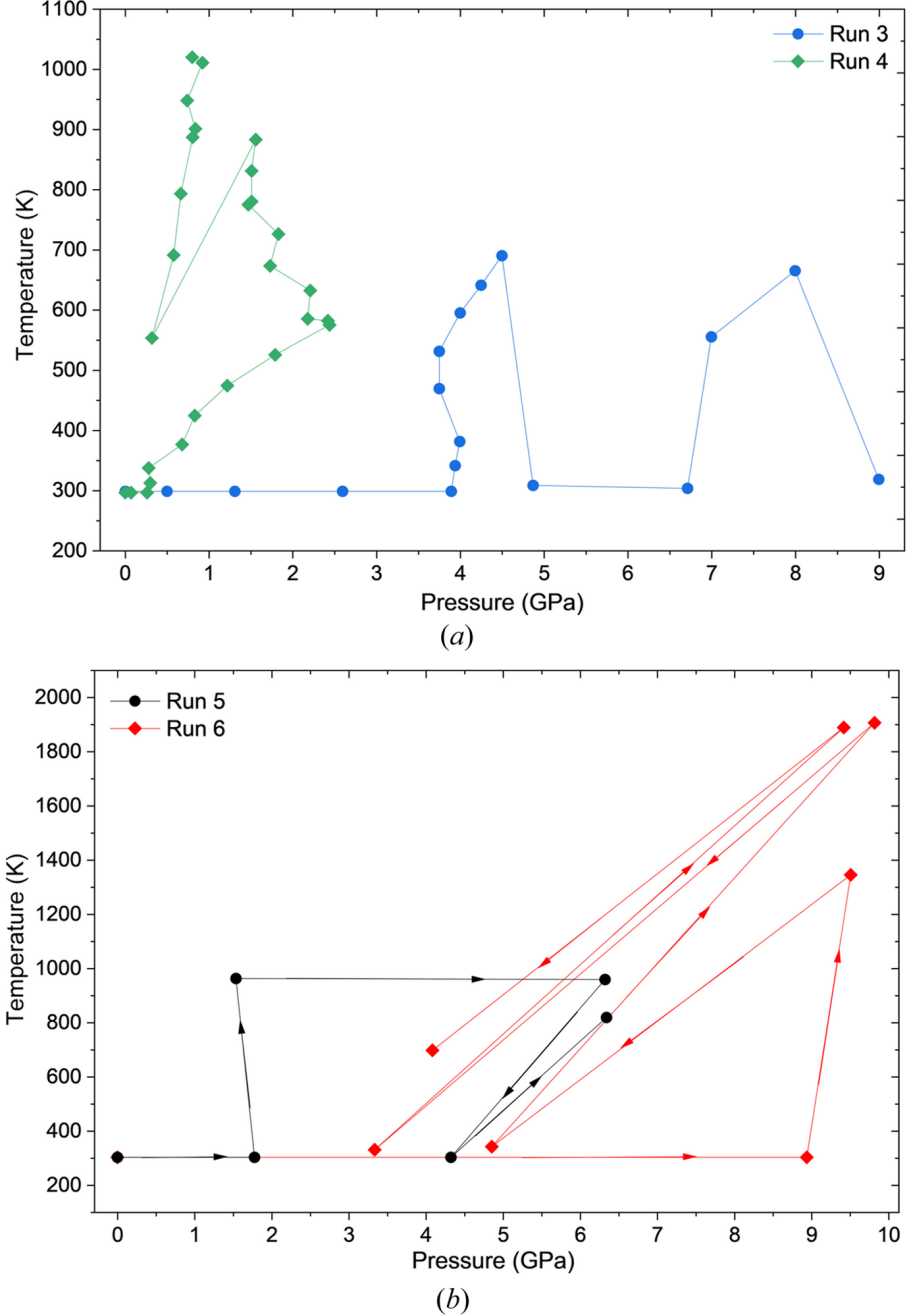
Pressure–temperature plots for (*a*) Run 3 and Run 4 and (*b*) Run 5 and Run 6.

High-pressure and high-temperature experiments were performed using an internal resistively heated Almax DAC equipped with 600 µm culet diamonds, Inconel gaskets and silicone oil as pressure medium. One experiment (Run 3) involved two high-temperature ramps, one around 4 GPa and a second one around 7 GPa. The maximum pressure reached was 9.00 (5) GPa, while the high-temperature ramps reached 690 K and 665 K, respectively [Fig. 1 ▸(*a*)]. Pressure was measured using samarium-doped SrB_4_O_7_ fluorescence while temperature was measured using a thermocouple placed in direct contact with one diamond. Data were collected at ID15b (ESRF, Grenoble, France) with a Mar555 detector and a wavelength of 0.41105 Å.

Another HP-HT experiment (Run 4) was conducted at the same beamline with an Eiger2X 9M CdTe detector [further details on the beamline setup given by Poręba *et al.* (2022 ▸)]. In this case, the maximum pressure reached was 2.44 (5) GPa and the maximum temperature 1020 K. Pressure was derived from the pressure–volume–temperature (*PVT*) equation of state (Angel *et al.*, 2017 ▸) of a quartz crystal placed in the cell along with the sample, while temperature was measured with a thermocouple. The pressure–temperature paths followed during the two experiments are given in Fig. 1 ▸(*a*), while information on the *P*/*T* conditions of the phase transitions is available as supporting information, S1.

SC-XRD data were indexed and integrated with *CrysAlis PRO RED* software (Rigaku Oxford Diffraction, 2019 ▸). For different pressure/temperature points, structural solution and refinement were done with *Jana2006* software (Petříček *et al.* 2014 ▸), using the *SuperFlip* charge-flipping algorithm (Palatinus & Chapuis 2007 ▸).

The last experiments (Run 5 and Run 6) were performed with the large-volume press (LVP) available at the ID06-LVP beamline (ESRF, Grenoble, France).

Both experiments used the standard 10/5 assembly. Previously dehydrated sodium carbonate crystals were finely ground along with platinum powder in an agate mortar and then pressed into a pellet of 2.4 mm diameter and 1.8 mm thickness. Sample and calibrant (platinum in Run 5, gold in Run 6) were placed directly into the graphite furnace to minimize the contribution of other materials to the resultant X-ray powder diffraction (XRPD) data pattern. The cylindrical sample was then inserted into a Cr-doped MgO octahedron with 10 mm-edge featuring two X-ray windows filled with boron epoxy rods. A detailed scheme of the assembly is available as supporting information, S2. The assembly was compressed using eight cubic WC anvils with a 5 mm truncation.

XRPD data were collected every 3 s on a PILATUS 3X CdTe 900K-W-ESRF 2D detector [details available in Crichton *et al.* (2023 ▸)] and a wavelength of 0.23262 Å for Run 5, 0.23393 Å for Run 6. Pressure and temperature conditions have been derived combining the press calibration (*i.e.* the relation between oil pressure and anvil pressure), and the equation of state of the sample and the calibrant. Pressure at high temperature has been obtained using the *PVT* equations of state of gold and platinum, respectively (Jamieson *et al.*, 1982 ▸). Due to calibration, pressure uncertainty in these runs reflects an error approximately one order of magnitude greater than that of the single-crystal experiments, further compounded by temperature uncertainties. As a result, these data do not provide accurate absolute *P*–*T* conditions, but all measurements are affected by the same systematic error. Consequently, this dataset remains reliable for constraining the relative behavior and topology of phase boundaries. The paths followed during experiments are reported in Fig. 1 ▸(*b*), while the *P*–*T* conditions of the phase transitions observed are reported as supporting information (S3 and S4). All XRPD patterns were automatically integrated at the beamline using detector geometry calibrated against a standard LaB_6_ reference sample. Selected patterns have been refined with the Rietveld method using *GSAS-EXPGUI* and *GSAS-II* (Toby & Von Dreele, 2013 ▸) based on the structural models obtained from the single-crystal experiments reported in this study (refinements are available as supporting information, S5).

## 3. Results and discussion

### 3.1. High-pressure experiments and equation of state

**Figure 2:**
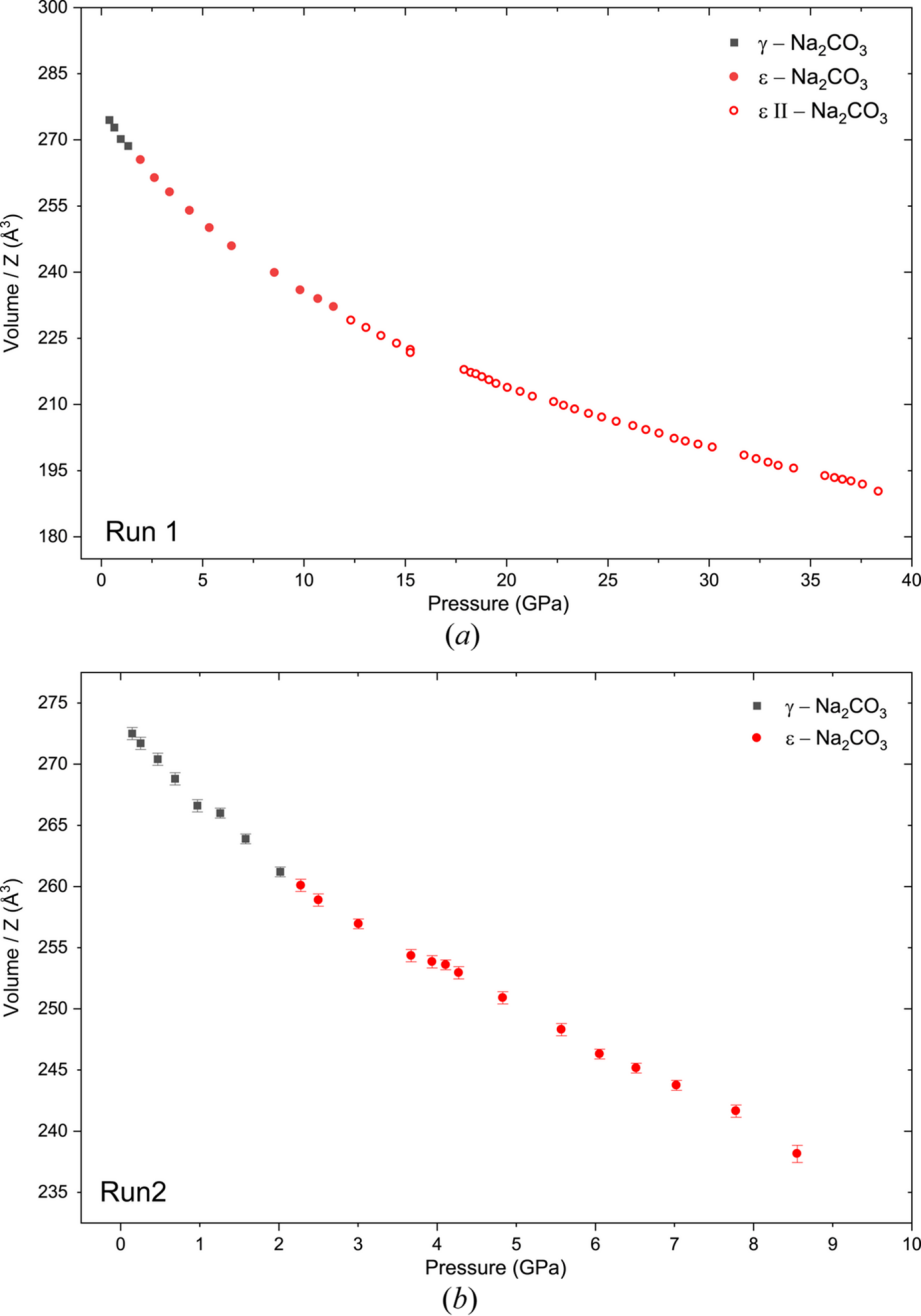
Volume variation with pressure observed in (*a*) Run 1 and (*b*) Run 2. Where not displayed, e.s.d. error bars are smaller than symbols.

The high-pressure experiments allowed the identification of new high-pressure polymorphs. Run 1 and Run 2 unit-cell parameters variation with pressure/temperature are reported in supporting information, S6 and S7, respectively. Experiment Run 1 showed two interesting pressure-induced phase transitions occurring at 1.33 (5) GPa and 11.46 (5) GPa. The two new structures, here referred to as ɛ and ɛ-II, are the result of second-order phase transitions, involving a slight re-arrangement of the structure and a variation in symmetry. This aspect will be discussed in detail in the next paragraph. Data collected during Run 1 [Fig. 2 ▸(*a*)] allowed the determination of the bulk modulus of the two new structures (Table 2 ▸). The same phase transition γ–ɛ has been observed in Run 2 [Fig. 2 ▸(*b*)], where data collected at low pressure allowed the determination of the bulk modulus of the γ phase. In experiment Run 2, silicone oil remains hydrostatic up to 4 GPa. In both experiments, volume–pressure data have been fitted using the *EoSFit7_GUI* program (Gonzalez-Platas *et al.*, 2016 ▸) with a second-order Birch–Murnaghan equation of state (Birch, 1947 ▸). The obtained parameters and refinement details are given in Table 2 ▸.

The second-order phase transition γ → ɛ does not have a significant effect on the compressibility of the structure, and the bulk modulus of the two polymorphs is not different within 3σ (Table 2 ▸). The compressibility of ɛ-II, in contrast, seems to be lower than the other structures, even though having only high-pressure data could result in an error on the bulk modulus estimation.

### 3.2. Crystal structures of ɛ and ɛ-II Na_2_CO_3_

**Figure 3:**
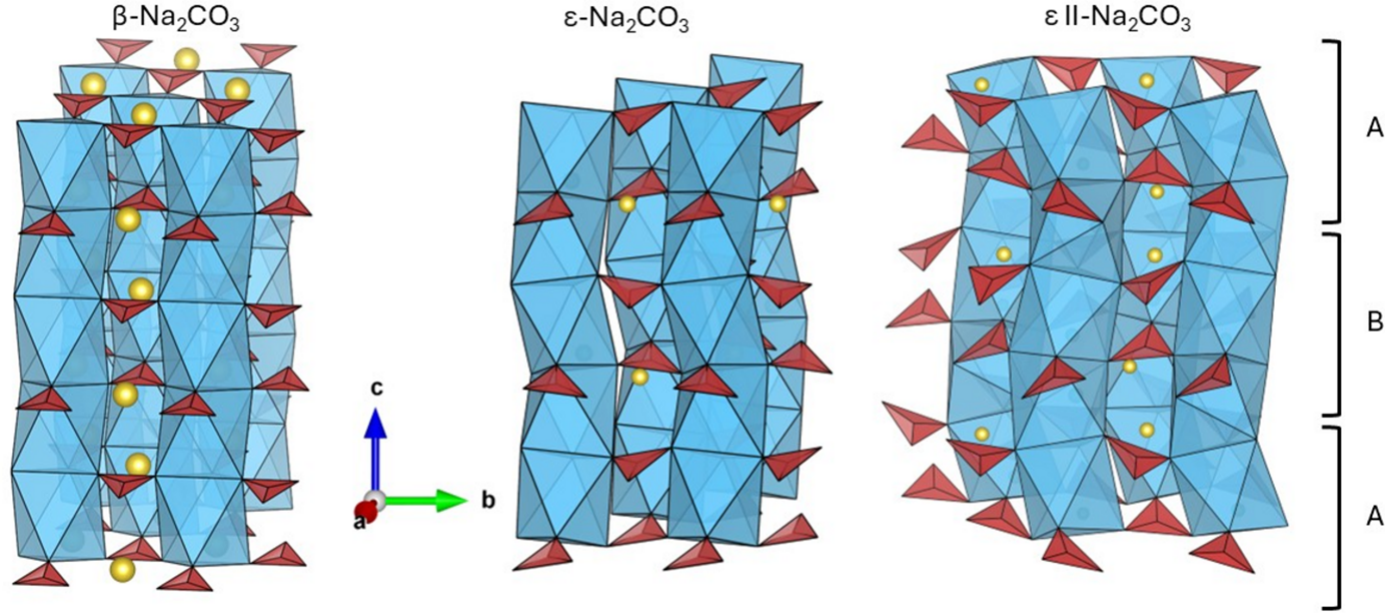
Schematic representation of the β structures. In blue Na polyhedral, in red CO_3_ groups and in yellow Na atoms.

As described by De Wolff & Tuinstra (1986 ▸), the sodium carbonate structure can be schematized as face-sharing columns of Na octahedra, parallel to the *c* axis, with Na and CO_3_ groups in between the columns. This representation is particularly efficient in underlining the relationship between the different structural configurations that this phase can have. Fig. 3 shows the structure of the β polymorph along with the two new structures, referred to as ɛ and ɛ-II. In Run 1, we observed a direct phase transition from the incommensurate structure γ to the ɛ structure, and then from ɛ to ɛ-II. The ɛ structure has been observed in all the experiments performed. ɛ-II, observed above 11.46 (5) GPa, is a distinct polymorph characterized by a triplication of the *b* axis (supporting information, S8). Unit-cell parameters and space groups of the different polymorphs are given in Table 3 ▸. While the β structure shows all CO_3_ groups in a co-planar distribution perpendicular to the *c* axis (Fig. 3 ▸), the ɛ structure can be described as the superimposition of two planar modules (*i.e.* A and B) with different orientation of CO_3_ groups. The tilting of the CO_3_ groups with respect to the *c* axis implies a deformation of the Na columns and a doubling of the unit-cell parameter *c*, as well as a reduction in symmetry from *C*2/*m* to *Cc*. The disorder in the CO_3_ groups orientation is even more evident in the ɛ-II structure, where the tilting varies also horizontally within the same planar unit, implying a tripled *b* unit-cell parameter. The atomic positions and refinement details of the two new structures are available in the supporting information (S9 and S10).

### 3.3. HP-HT experiments and phase diagram

**Figure 4:**
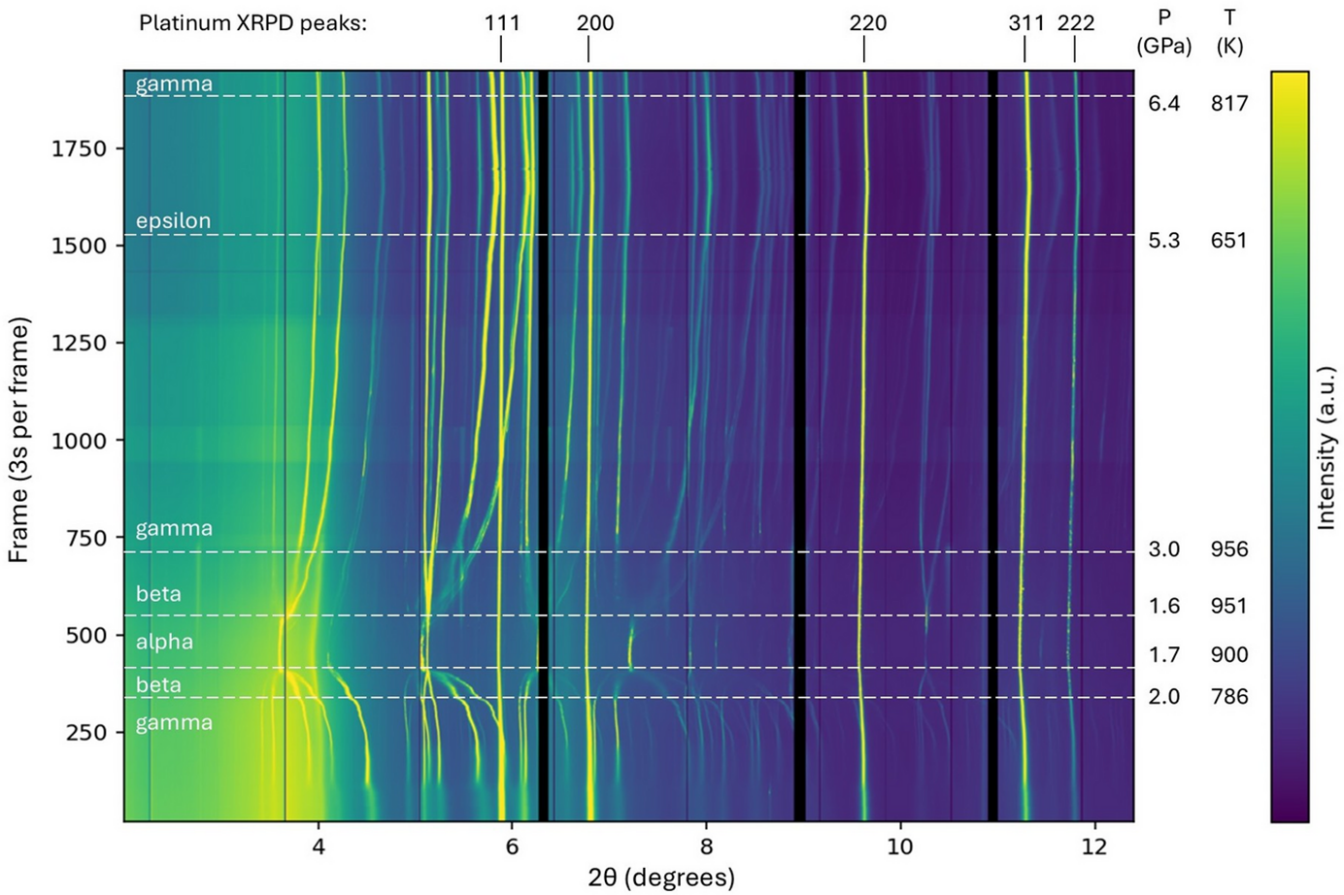
XRPD peaks position variation in time in Run 5. Vertical axis shows the frame number, each frame has been collected for 3 s. *P* and *T* are estimated following the procedure described in *Methods*[Sec sec2] and are subject to significant uncertainty. The black areas represent filters applied to the MgO peak positions to enhance the visualization of the sample contribution.

SC-XRD has been performed in experiments Run 3 and Run 4. Specifically, Run 3 confirmed the stability of the ɛ phase even under high-temperature conditions, while Run 4, performed at relatively low-pressure conditions [*i.e.* up to 2.44 (5) GPa], allowed the identification of the γ–β–α phase transitions with temperature. These experiments served as accurate and precise anchor points in the building of the phase diagram. Variation of unit-cell parameters with pressure/temperature for Run 3 and Run 4 are given in S11 and S12 of the supporting information, respectively. *In situ* MA XRPD experiments (Run 5 and Run 6) allowed the identification of the same phase transitions at different pressure–temperature conditions (supporting information, S3 and S4). Fig. 4 ▸ shows the variation of the XRPD peaks position during *P* and *T* variations in Run 5.

**Figure 5:**
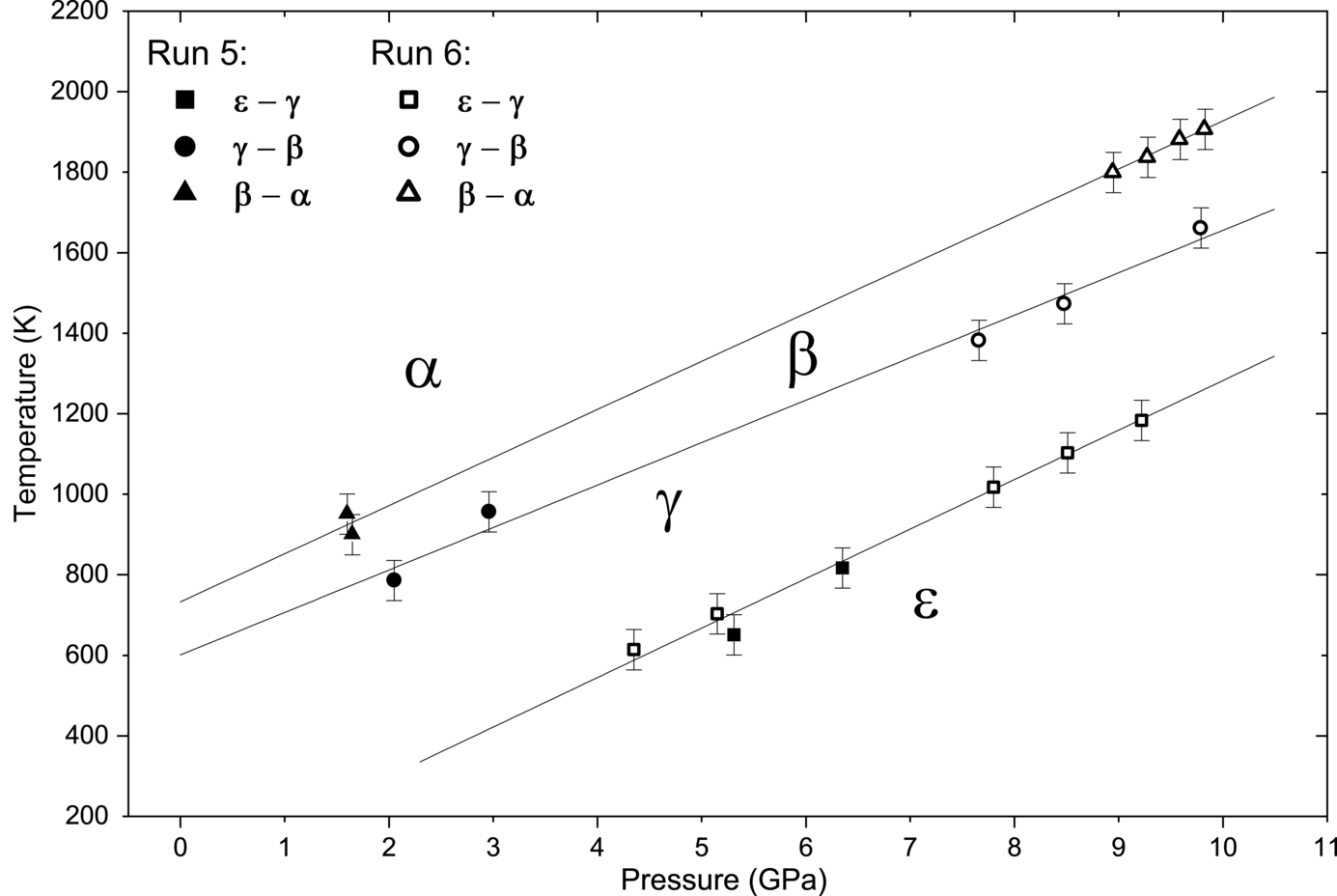
Experimental points obtained in Run 5 and Run 6. We report an estimated error on temperature of ±50 K. Linear trendline of data is shown to assess the relative orientation of the phase boundaries.

The phase transitions and their reversibility observed in LVP experiments (Fig. 5 ▸) further constrained the topology of the phase boundaries.

## 4. Conclusions

**Figure 6:**
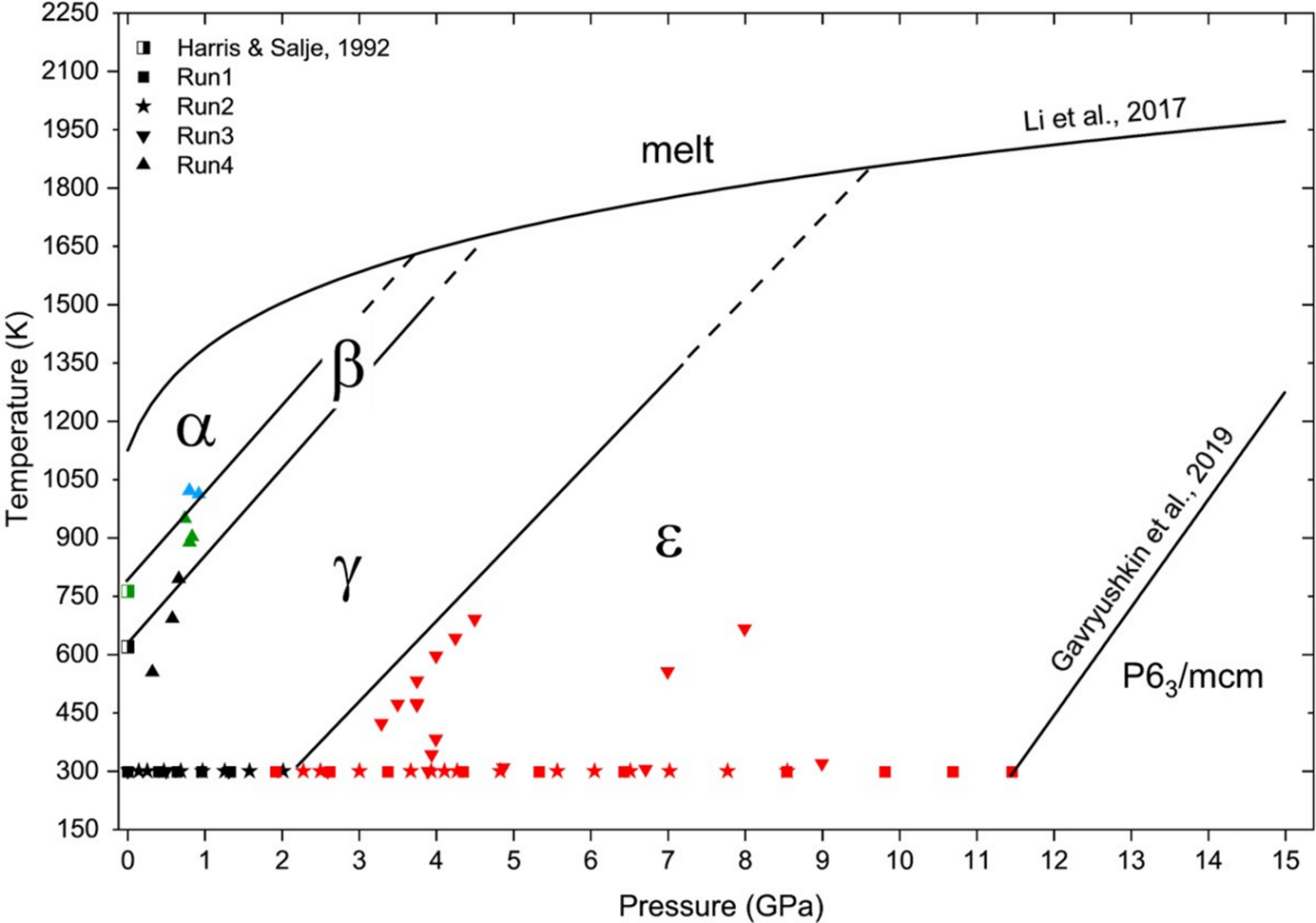
Phase diagram of Na_2_CO_3_ obtained from experimental data. Symbols represent the different experimental runs as reported in the legend, colors represent the different polymorphs (α in blue, β in green, γ in black, ɛ in red).

We report two new high-pressure sodium carbonate structures, ɛ and ɛ-II. The ɛ polymorph is characterized by a doubling of the *c* unit-cell parameter compared to the γ and β polymorphs, and crystallizes in space group *Cc*. Its bulk modulus is 47.6 (8) GPa and is not significantly different from that of the γ phase. Its stability is confirmed by *in situ* single-crystal experiments and *in situ* LVP experiments. By exploring a range of pressure–temperature conditions, we observed multiple phase transitions and their reversibility, allowing us to constrain the topology of the Na_2_CO_3_ phase diagram. Using experimental single-crystal data as precise and accurate fixed points, and combining them with the sub-parallel phase-boundary topology observed in LVP experiments, we propose an updated phase diagram for sodium carbonate (Fig. 6 ▸). Interestingly, our data also align well with the phase boundary reported by Gavryushkin *et al.* (2019 ▸) for the hexagonal *P*6_3_/*mcm* polymorph, stable above 12 GPa. The high-pressure stability limit of the ɛ phase observed in this study is consistent with the phase transition to the hexagonal polymorph reported by Gavryushkin *et al.* (2019 ▸). The ɛ-II structure, observed above 11.46 (5) GPa may represent a low-temperature polymorph or a metastable phase of Na_2_CO_3_.

Compared with the literature (Shatskiy *et al.*, 2015 ▸), we observe a widening of the incommensurate γ-phase stability field toward higher pressures and temperatures. Both single-crystal and LVP experiments clearly show a positive *P*–*T* slope for the γ → β → α transition sequence.

Our results also show a wide stability field for the ɛ polymorph at pressures and temperatures compatible with the Earth’s mantle. These results provide robust experimental benchmarks that improve the reliability of the phase diagram and clarify the relationships between existing and newly identified phases. The updated phase diagram offers a more complete framework for interpreting the structural evolution of sodium carbonate under pressure and temperature, and provides essential constraints for future experimental, computational and geological studies.

## Supplementary Material

Crystal structure: contains datablock(s) global, epsilon-Na2CO3_P5, epsilonII-Na2CO3_P16. DOI: 10.1107/S2052520626004956/dk5146sup1.cif

Structure factors: contains datablock(s) epsilon-Na2CO3_P5, (epsilon-Na2CO3_P5). DOI: 10.1107/S2052520626004956/dk5146epsilon-Na2CO3_P5sup2.hkl

Structure factors: contains datablock(s) epsilonII-Na2CO3_P16, (epsilonII-Na2CO3_P16). DOI: 10.1107/S2052520626004956/dk5146epsilonII-Na2CO3_P16sup3.hkl

Supporting Figures and Tables. DOI: 10.1107/S2052520626004956/dk5146sup4.pdf

CCDC references: 2553748, 2553749

## Figures and Tables

**Table 1:** Experimental details for different runs

Run	Setup	Pressure medium	*P* (GPa)	*T* (K)	Beamline
1	DAC	Helium	0–38.37 (5)	RT	ID15b, ESRF
2	DAC	Silicone oil	0–8.55 (5)	RT	ID09, ESRF
3	Resistively heated DAC	Silicone oil	Fig. 1 ▸(*a*)		ID15b, ESRF
4	Resistively heated DAC	Silicone oil	Fig. 1 ▸(*a*)		ID15b, ESRF
5	LVP	–	Fig. 1 ▸(*b*)		ID06-LVP, ESRF
6	LVP	–	Fig. 1 ▸(*b*)		ID06-LVP, ESRF

**Table 2:** Equation of state parameters obtained by fit

Run	Phase	*V*_0_	*K*_0_	maxΔ*P*	_w_χ^2^
2	γ	273.4 (4)	41 (2)	−0.11	0.4
1	ɛ	549.7 (9)	47.6 (8)	0.20	2.52
1	ɛ-II	1597 (5)	59.8 (12)	1.40	26.20

**Table 3:** Unit-cell parameters and space groups of different Na_2_CO_3_ polymorphs Data from this study are obtained by single-crystal XRD.

Phase	*a* (Å)	*b* (Å)	*c* (Å)	β (°)	Volume (Å^3^)	Space group	*P* (GPa)	*T* (K)	Source
β	9.013 (2)	5.237 (2)	6.321 (2)	96.83 (2)	295.82	*C*2/*m*	0	673	de Pater (1979 ▸)
ɛ	8.832 (5)	5.2024 (5)	11.8155 (13)	102.03 (5)	531.0 (3)	*Cc*	1.92 (5)	300	This study, Run 1
ɛ-II	8.526 (3)	14.9693 (1)	11.0872 (1)	103.75 (4)	1374.5 (5)	*Cc*	12.32 (5)	300	This study, Run 1
