# Supplementary material for: High-pressure and high-temperature polymorphism of Na2CO3 up to 10 GPa
Source: Acta Crystallogr B Struct Sci Cryst Eng Mater. 2026 Jun 23;82(Pt 4):410–5. doi: 10.1107/S2052520626004956 (PMC13470794; doi:10.1107/S2052520626004956)
Supplement: Supplementary file 1 [file b-82-00410-sup4.pdf]

STRUCTURAL SCIENCE
CRYSTAL ENGINEERING
MATERIALS

Volume 82 (2026)

Supporting information for article:

High-pressure and high-temperature polymorphism of Na2CO3 up to 10 GPa

Benedetta Chrappan Soldavini, Marco Merlini, Chiara Mangano, Davide Comboni, Wilson Crichton and Michael Hanfland

Supplementary Material

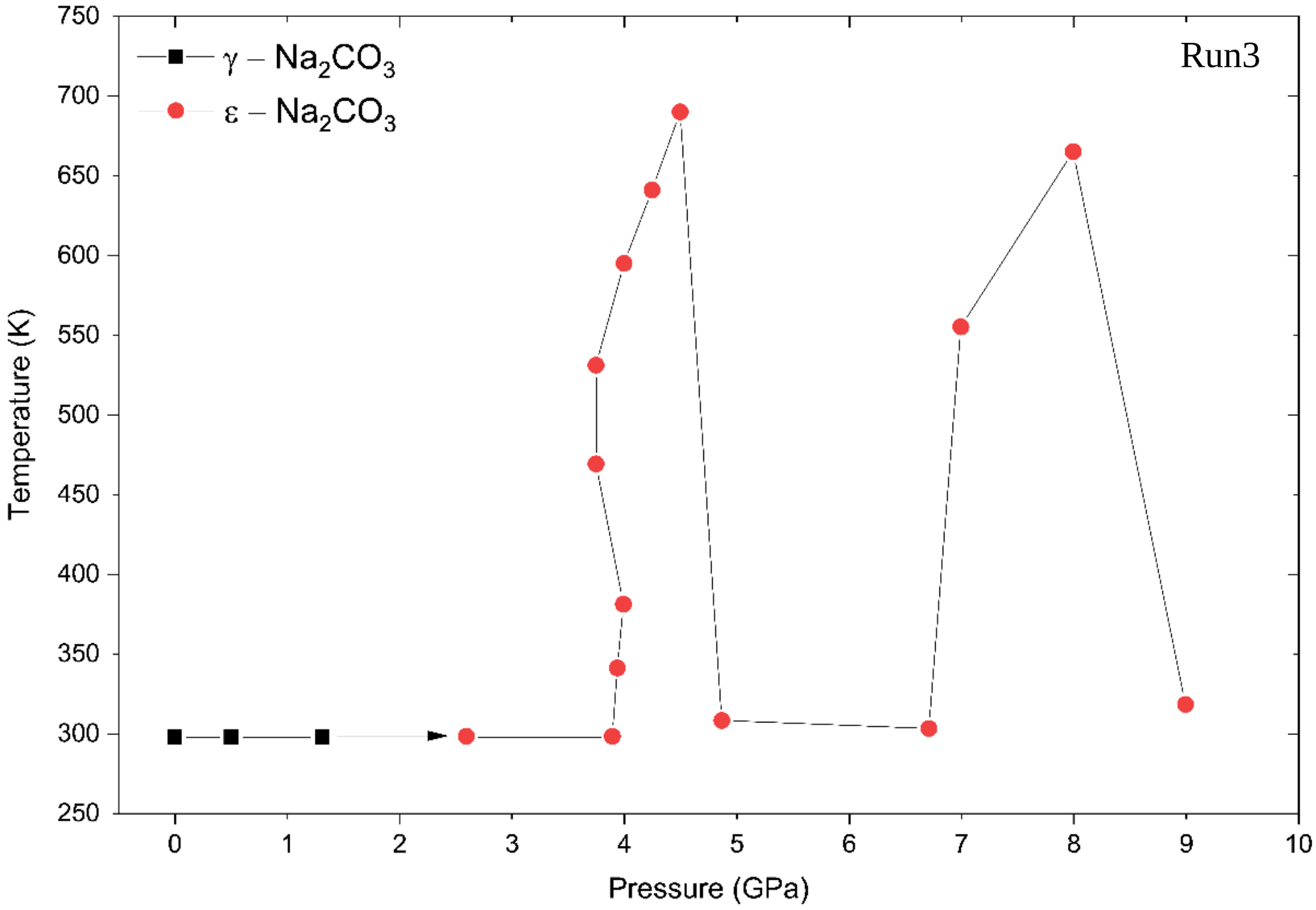

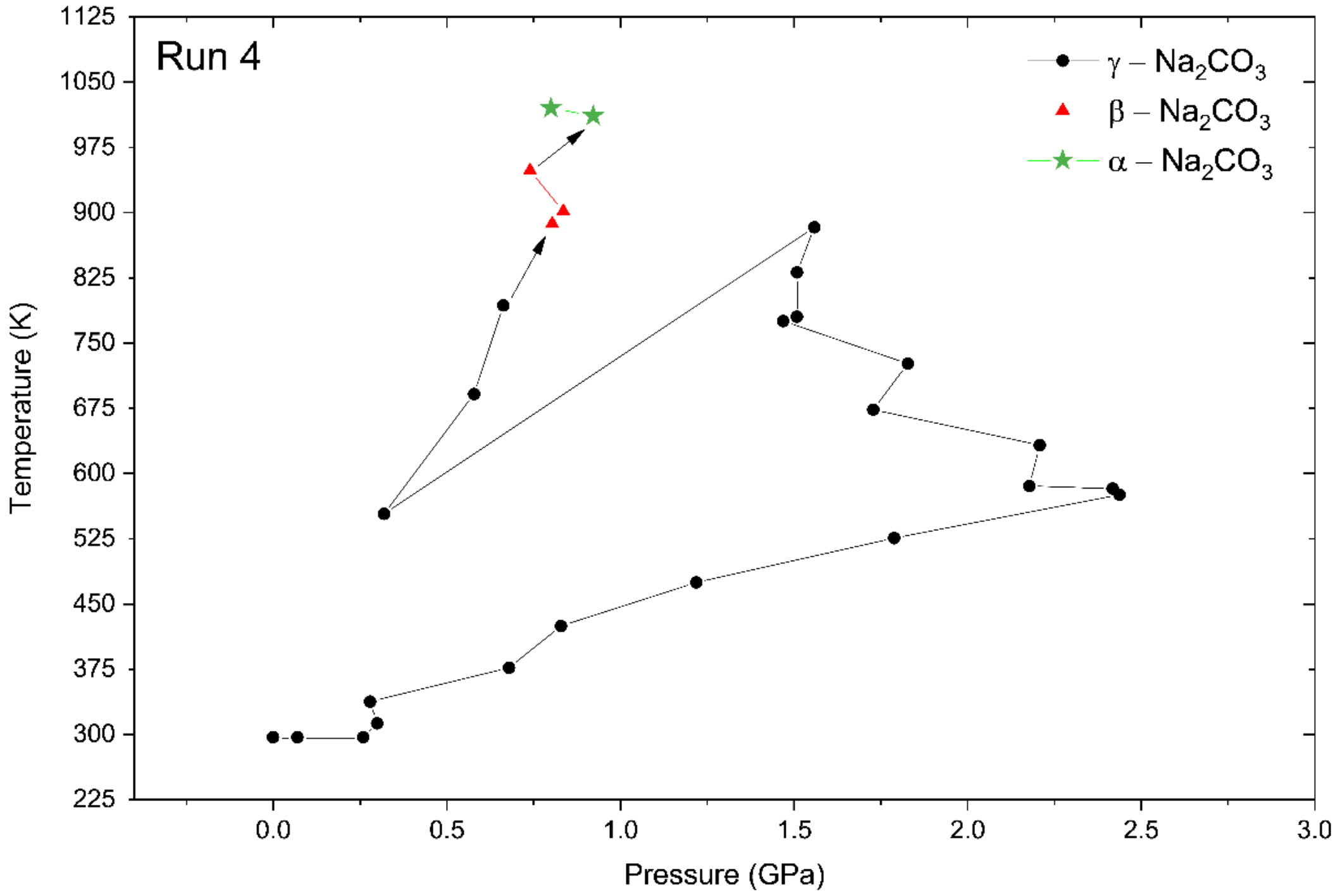

S1: P-T path followed in single-crystal in-situ experiments Run3 and Run4.

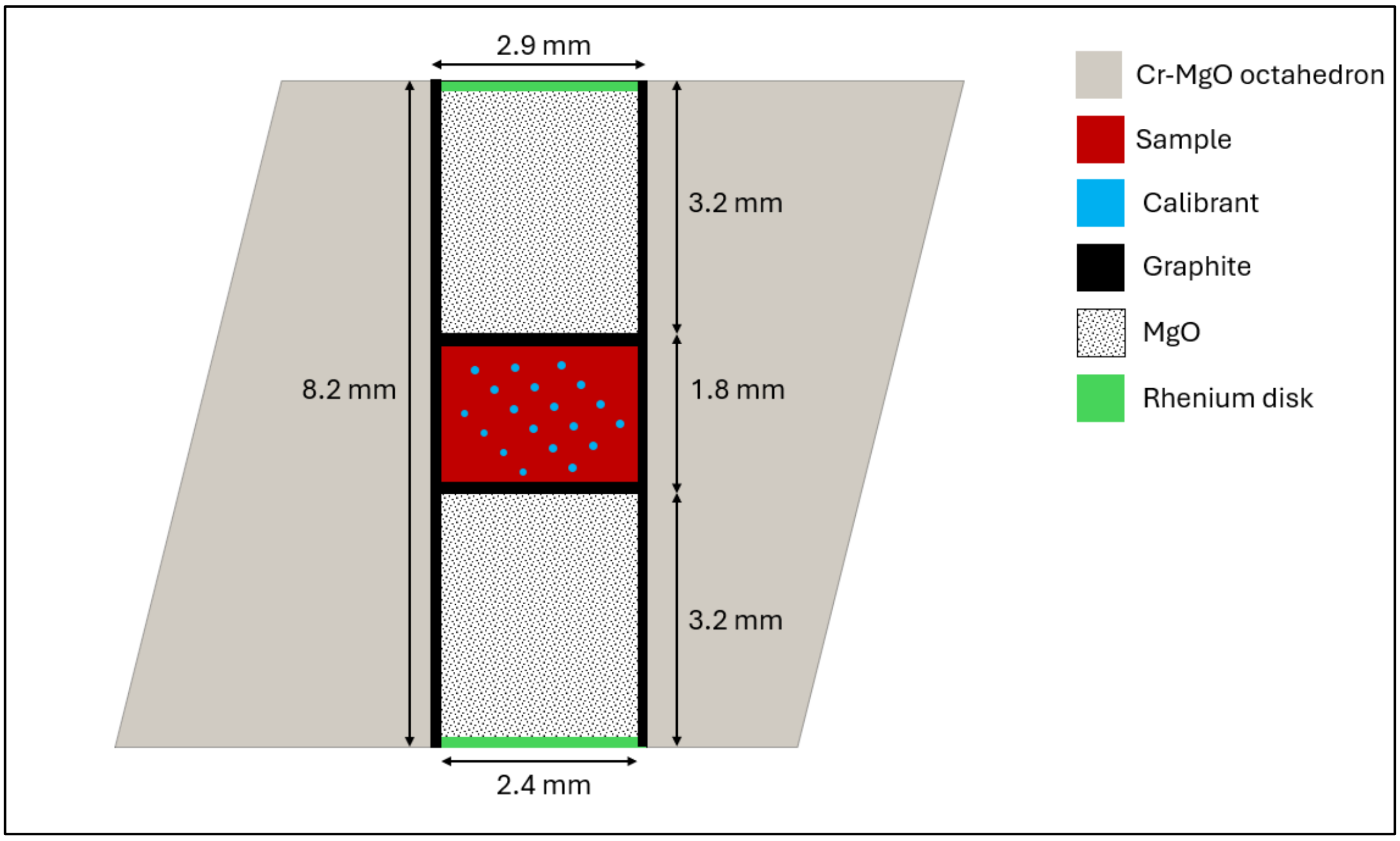

S2: Assebly 10/5 used for Multi Anvil experiments (Run5 and Run6) at ID06-LVP beamline (ESRF, Grenoble).

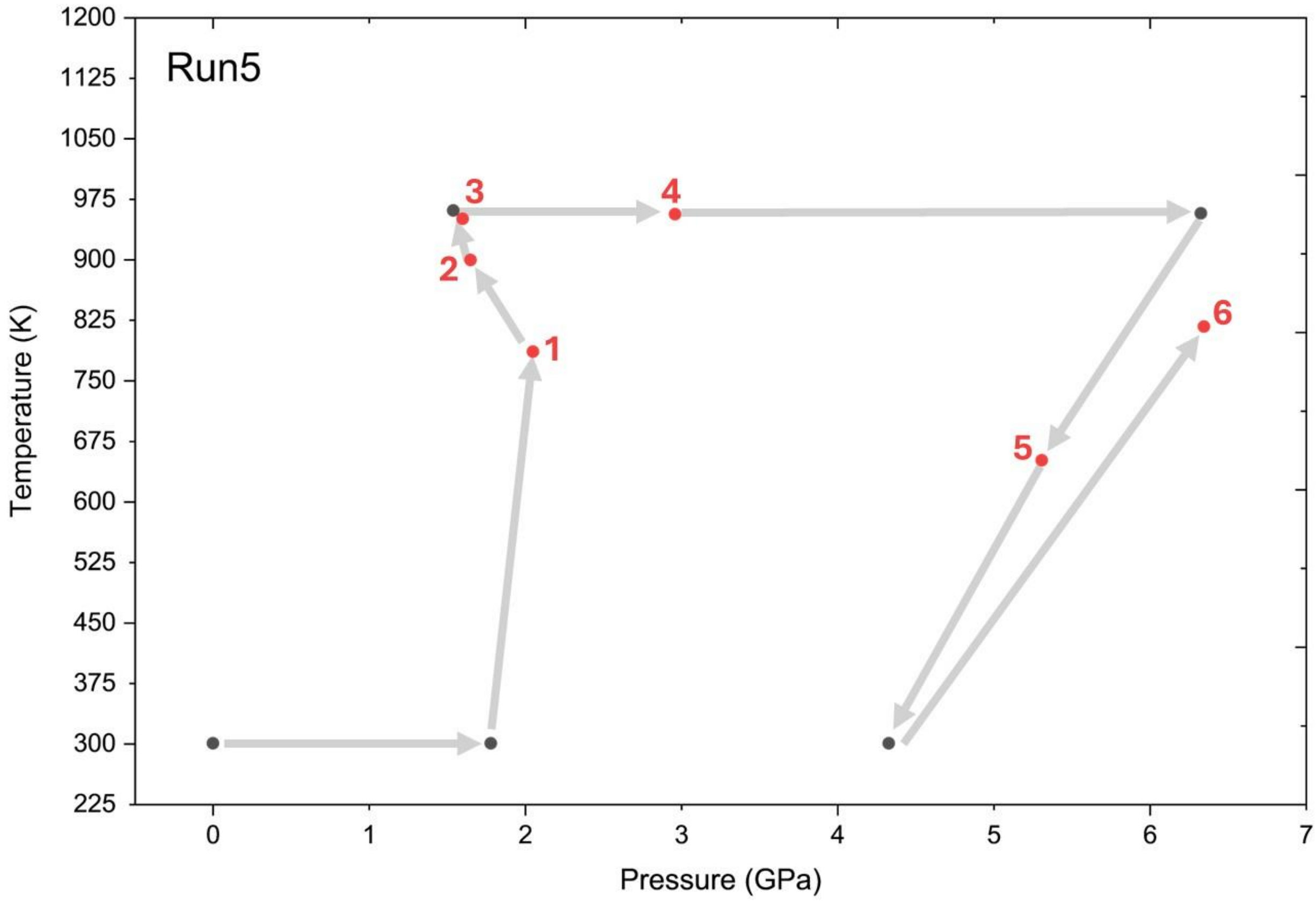

S3: Run5 PT path followed during experiment. In red the transitions observed: 1 γ-β, 2 β-α, 3 α-β, 4 β-γ, 5 γ-ε, 6 ε-γ.

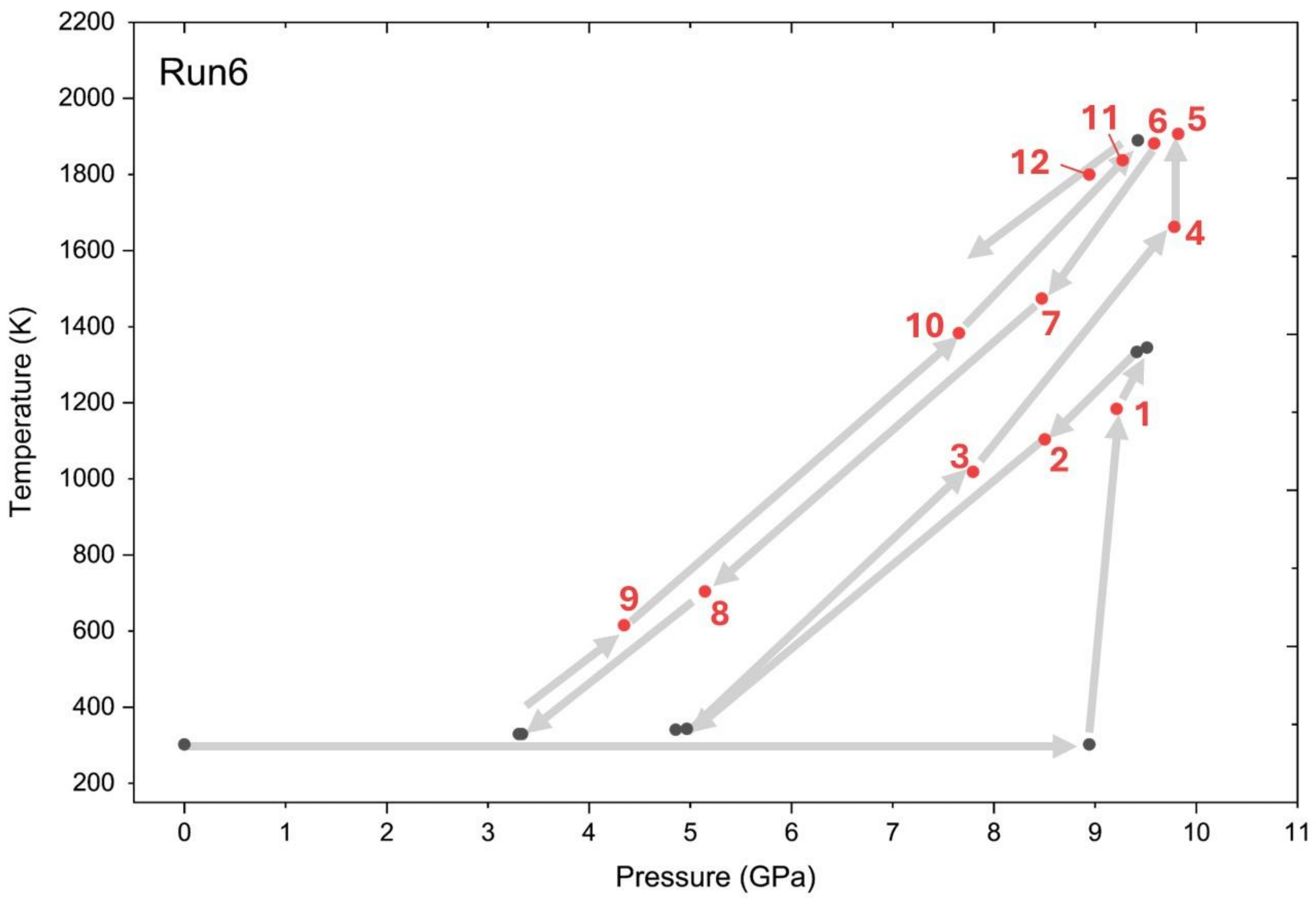

S4: Run6 PT path followed during experiment. In red the transitions observed: 1 ε-γ, 2 γ-ε, 3 ε-γ, 4 γ-β, 5 β-α, 6 α-β, 7 β-γ, 8 γ-ε, 9 ε-γ, 10 γ-β, 11 β-α, 12 α-β.

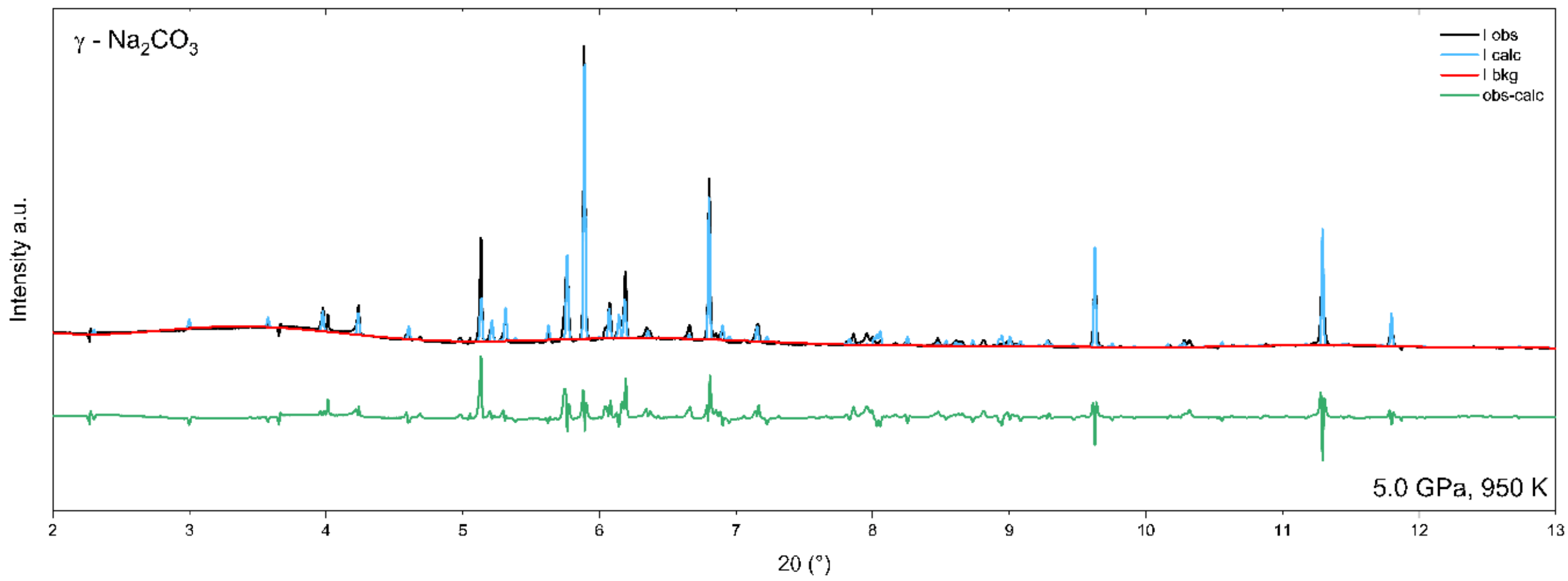

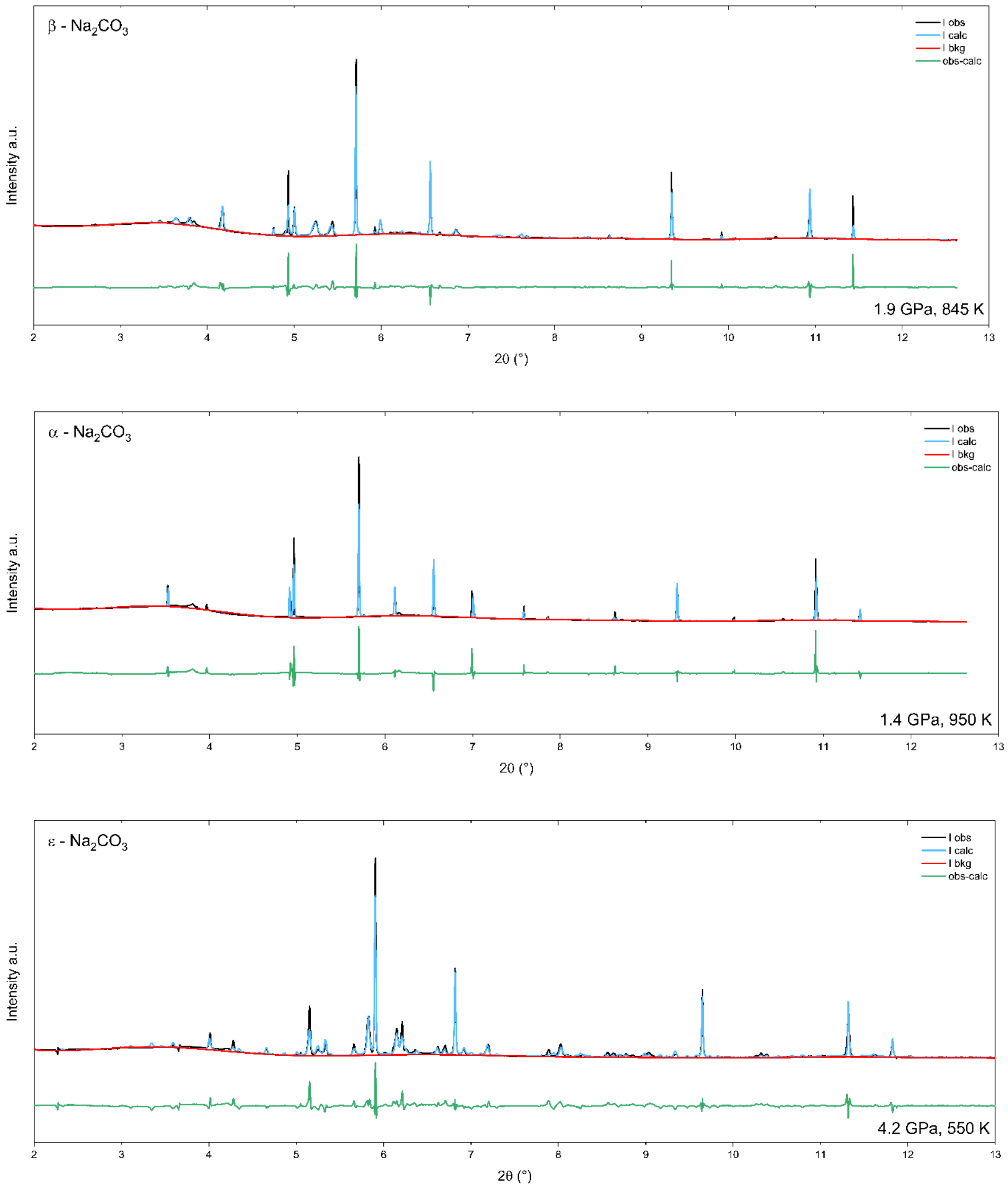

S5: Selected XRPD from Run 5. The γ structure has been refined using the average monoclinic non-incommensurate structure, but satellite peaks are present in the pattern. The intensities are not always matching due to large crystal domains forming during experiments, resulting in intense spots measured on the detector.

Step	P (GPa)	a (Å)	b (Å)	c (Å)	B (°)	Volume (Å³)	Phase
1	0.40(5)	8.94(3)	5.203(10)	6.02(5)		274.5(8)	γ
2	0.64(5)	8.877(11)	5.2319(13)	6.0003(19)		272.8(4)	γ
3	0.96(5)	8.855(9)	5.217(5)	5.979(2)		270.2(4)	γ
4	1.33(5)	8.845(5)	5.220(3)	5.9517(13)		268.6(3)	γ
5	1.92(5)	8.832(5)	5.2024(5)	11.8155(13)		531.0(4)	ε
6	2.62(5)	8.798(6)	5.1867(6)	11.7295(12)		522.8(4)	ε
7	3.37(5)	8.776(6)	5.1731(6)	11.6551(15)		516.4(4)	ε
8	4.35(5)	8.739(6)	5.1556(5)	11.5647(14)		508.0(4)	ε
9	5.33(5)	8.701(6)	5.1389(5)	11.4854(14)		500.1(4)	ε
10	6.43(5)	8.658(5)	5.1204(5)	11.4080(13)		491.9(3)	ε
11	8.54(5)	8.610(6)	5.0881(8)	11.278(2)		479.8(4)	ε
12	9.81(5)	8.560(6)	5.0708(7)	11.2085(19)		471.9(4)	ε
13	10.69(5)	8.539(6)	5.0604(7)	11.1704(19)		467.9(4)	ε
14	11.46(5)	8.532(7)	5.0441(13)	11.132(3)		464.3(5)	ε
15	12.09(5)	8.510(10)	15.035(3)	11.085(3)		1375.0(1)	ε II
16	12.32(5)	8.526(3)	14.9693(10)	11.0872(10)		1374.4(6)	ε II
17	13.07(5)	8.519(3)	14.9027(8)	11.0581(9)		1364.4(6)	ε II
18	13.81(5)	8.506(3)	14.8505(14)	11.0236(12)		1353.4(6)	ε II
19	14.58(5)	8.495(3)	14.7944(10)	10.9905(10)		1342.9(6)	ε II
20	15.26(5)	8.481(3)	14.7654(12)	10.9661(12)		1334.5(5)	ε II
21	17.91(5)	8.440(3)	14.6561(9)	10.8766(10)		1307.1(6)	ε II
22	18.24(5)	8.435(3)	14.6404(4)	10.8643(10)		1303.2(6)	ε II
23	18.49(5)	8.433(3)	14.6329(17)	10.8577(11)		1301.3(6)	ε II
24	18.79(5)	8.425(3)	14.6165(8)	10.8456(9)		1297.3(6)	ε II
25	19.14(5)	8.419(3)	14.6016(8)	10.8329(9)		1293.3(6)	ε II
26	19.49(5)	8.407(3)	14.5861(16)	10.8212(11)		1288.3(6)	ε II
27	20.05(5)	8.397(3)	14.5647(17)	10.8036(11)		1282.7(6)	ε II
28	20.68(5)	8.392(3)	14.5400(7)	10.7825(8)		1277.3(6)	ε II
29	21.29(5)	8.377(3)	14.5194(17)	10.7664(11)		1270.8(6)	ε II
30	22.34(5)	8.366(3)	14.4906(17)	10.742(11)		1263.4(6)	ε II
31	22.83(5)	8.360(3)	14.4691(8)	10.7229(9)		1258.5(6)	ε II
32	23.37(5)	8.352(3)	14.4493(9)	10.7075(10)		1253.5(6)	ε II
33	24.06(5)	8.342(3)	14.4258(8)	10.6873(9)		1247.4(6)	ε II
34	24.71(5)	8.333(2)	14.4077(7)	10.6711(8)		1242.3(6)	ε II
35	25.43(5)	8.323(3)	14.3871(8)	10.6527(9)		1236.5(6)	ε II
36	26.25(5)	8.312(3)	14.3657(8)	10.6336(8)		1230.7(6)	ε II
37	26.89(5)	8.303(3)	14.3417(9)	10.6172(10)		1225.3(6)	ε II
38	27.54(5)	8.297(3)	14.3212(9)	10.6021(11)		1220.6(6)	ε II
39	28.29(5)	8.287(3)	14.2923(9)	10.5766(10)		1213.4(6)	ε II
40	28.84(5)	8.280(3)	14.2780(8)	10.5642(9)		1209.6(6)	ε II
41	29.47(5)	8.269(3)	14.2654(8)	10.5530(9)		1205.5(6)	ε II
42	30.17(5)	8.264(3)	14.2501(16)	10.5322(10)		1201.5(5)	ε II
43	31.74(5)	8.243(3)	14.2074(15)	10.5029(9)		1190.5(5)	ε II
44	32.34(5)	8.233(3)	14.1904(16)	10.4882(9)		1185.7(5)	ε II
45	32.93(5)	8.220(3)	14.1748(15)	10.4747(8)		1181.0(5)	ε II
46	33.43(5)	8.214(3)	14.1578(16)	10.4611(9)		1176.7(5)	ε II

47	34.19(5)	8.208(3)	14.1428(17)	10.4465(9)		1172.9(5)	ε II
48	35.73(5)	8.185(2)	14.1104(7)	10.4138(8)		1162.6(5)	ε II
49	36.21(5)	8.179(3)	14.0995(16)	10.4074(9)		1159.9(5)	ε II
50	36.60(5)	8.174(3)	14.0899(16)	10.4008(8)		1157.6(5)	ε II
51	37.02(5)	8.172(3)	14.0793(17)	10.3925(9)		1155.4(5)	ε II
52	37.59(5)	8.163(3)	14.0623(16)	10.3778(8)		1150.9(5)	ε II
53	38.37(5)	8.139(3)	14.0287(14)	10.3502(7)		1141.5(4)	ε II

S6: Unit cell and volume variation with pressure in Run1. Esds in parentheses.

**Step**	**P (GPa)**	**a (Å)**	**b (Å)**	**c (Å)**	**Β (°)**	**Volume (Å^3)**	**Phase**
1	0.15(5)	8.865(7)	5.2236(14)	6.0054(11)	101.48(3)	272.5(5)	γ
2	0.25(5)	8.854(8)	5.2223(16)	5.9966(12)	101.53(4)	271.7(5)	γ
3	0.47(5)	8.845(9)	5.2203(15)	5.9779(14)	101.60(4)	270.4(5)	γ
4	0.69(5)	8.844(7)	5.2102(13)	5.9579(12)	101.71(3)	268.8(5)	γ
5	0.97(5)	8.833(10)	5.1989(18)	5.9332(15)	101.85(5)	266.6(5)	γ
6	1.26(5)	8.822(10)	5.2035(17)	5.9246(15)	101.98(5)	266.0(4)	γ
7	1.58(5)	8.806(9)	5.1976(16)	5.8973(15)	102.14(4)	263.9(4)	γ
8	2.02(5)	8.782(9)	5.1871(16)	5.8659(16)	102.20(5)	261.2(4)	γ
9	2.28(5)	8.783(5)	5.1789(8)	11.699(2)	101.19(3)	520.2(5)	ε
10	2.50(5)	8.762(4)	5.1760(7)	11.6873(16)	102.34(2)	517.8(5)	ε
11	3.01(5)	8.733(6)	5.1709(8)	11.655(2)	102.47(3)	513.9(4)	ε
12	3.67(5)	8.710(6)	5.1582(9)	11.609(2)	102.76(3)	508.7(5)	ε
13	3.94(5)	8.709(5)	5.1598(7)	11.5867(17)	102.79(3)	507.7(5)	ε
14	4.11(5)	8.711(4)	5.1568(5)	11.5834(12)	102.893(19)	507.2(4)	ε
15	4.27(5)	8.709(6)	5.1526(9)	11.569(2)	102.94(3)	505.9(5)	ε
16	4.83(5)	8.682(6)	5.1469(8)	11.5288(18)	103.11(3)	501.8(5)	ε
17	5.57(5)	8.661(3)	5.1380(5)	11.4745(12)	103.462(18)	496.6(5)	ε
18	6.05(5)	8.627(7)	5.1327(10)	11.444(2)	103.57(4)	492.6(4)	ε
19	6.52(5)	8.626(5)	5.1256(6)	11.4151(16)	103.71(2)	490.3(4)	ε
20	7.03(5)	8.613(6)	5.1192(8)	11.386(2)	103.85(3)	487.5(4)	ε
21	7.78(5)	8.596(9)	5.1125(10)	11.337(3)	104.05(5)	483.3(5)	ε
22	8.55(5)	8.563(13)	5.0937(14)	11.266(4)	104.24(7)	476.3(7)	ε

S7: Unit cell and volume variation with pressure in Run2. Esds in parentheses.

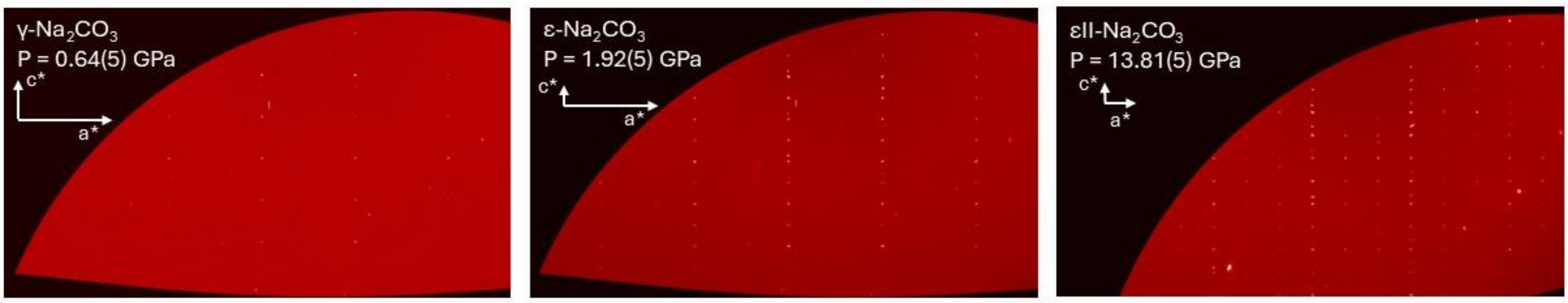

S8: 1kl reciprocal space reconstruction of the different polymorphs. Additional peaks from a second smaller crystal are also present.

Site label	Site type symbol	x	y	z	Site adp type	U_{iso}	Symmetry multiplicity	s.o.f.	Wyckoff position
Na1	Na	0.4984(13)	0.0404(10)	0.2194(4)	Uiso	0.0235(13)	4	1	d
Na2	Na	0.1604(17)	0.0072(10)	0.0907(5)	Uiso	0.0173(5)	4	1	d
Na4	Na	0.6657(15)	-0.4588(4)	0.3422(5)	Uiso	0.0168(5)	4	1	d
O2	O	0.090(2)	-0.3357(17)	-0.0370(8)	Uiso	0.0221(17)	4	1	d
O3	O	0.294(2)	-0.2601(15)	0.2547(6)	Uiso	0.023(2)	4	1	d
O4	O	0.3827(18)	0.0156(13)	0.0081(6)	Uiso	0.0214(18)	4	1	d
Na3	Na	0.8368(15)	0.0374(10)	0.4642(4)	Uiso	0.0266(14)	4	1	d
O5	O	0.539(2)	0.2520(13)	0.4331(6)	Uiso	0.0185(18)	4	1	d
O6	O	0.235(2)	-0.6623(18)	0.2153(8)	Uiso	0.027(2)	4	1	d
O7	O	0.4585(17)	-0.4878(13)	0.1796(6)	Uiso	0.0196(18)	4	1	d
C1	C	0.002(2)	-0.5136(16)	-0.0320(7)	Uiso	0.0090(18)	4	1	d
C2	C	0.329(2)	-0.4625(19)	0.2157(7)	Uiso	0.013(2)	4	1	d

**Refinement Details**	
Phase	ε
Chemical formula	Na_2CO_3
Crystal system	Monoclinic
Space group	Cc
a (P = 1.92(5) GPa)	8.832(5) Å
b (P = 1.92(5) GPa)	5.2024(5) Å
c (P = 1.92(5) GPa)	11.8155(13) Å
β	102.03(5)°
Cell volume (P = 1.92(5) GPa)	531.0(3) Å³
Z	8
Density (P = 3 GPa)	2.65 g/cm³
Total number of reflections	411
No. reflections I > 3σ(I)	358
R1	0.0511
R1 (all)	0.0612

wR	0.0600
wR (all)	0.0608
Number of parameters	49
GooF	4.09

S9: ε atomic positions and refinement details

Site label	Site type symbol	x	y	z	Site adp type	U_{iso}	Symmetry multiplicity	s.o.f.	Wyckoff position
Na1	Na	0.9972(15)	0.3547(3)	0.9865(6)	Uiso	0.0177(11)	4	1	d
Na2	Na	0.3158(13)	0.3601(4)	0.8588(5)	Uiso	0.0225(12)	4	1	d
Na3	Na	0.8103(13)	0.5085(4)	0.8698(5)	Uiso	0.0249(14)	4	1	d
Na4	Na	0.7999(14)	0.1861(3)	0.8708(6)	Uiso	0.0251(14)	4	1	d
Na5	Na	0.4675(14)	0.1783(4)	0.2360(5)	Uiso	0.0235(11)	4	1	d
Na6	Na	0.4614(16)	0.1909(3)	0.9854(6)	Uiso	0.0212(12)	4	1	d
C1	C	0.646(3)	0.3474(5)	0.8608(10)	Uiso	0.0015(19)	4	1	d
O1	O	0.548(2)	0.2783(6)	0.8584(9)	Uiso	0.025(2)	4	1	d
C2	C	0.653(3)	0.6794(6)	0.8664(10)	Uiso	0.005(2)	4	1	d
O2	O	0.840(2)	0.5900(5)	1.0721(8)	Uiso	0.0166(18)	4	1	d
O3	O	0.424(2)	0.2978(5)	0.1176(9)	Uiso	0.031(2)	4	1	d
O4	O	0.260(2)	0.5127(5)	0.8146(7)	Uiso	0.0196(19)	4	1	d
O5	O	0.2976(19)	0.4224(5)	1.0582(7)	Uiso	0.0118(16)	4	1	d
Na7	Na	0.4613(15)	0.5187(3)	0.9917(5)	Uiso	0.0167(11)	4	1	d
O6	O	0.694(2)	0.8215(5)	1.1633(8)	Uiso	0.0194(19)	4	1	d
C3	C	0.145(3)	0.5147(6)	0.8576(11)	Uiso	0.008(2)	4	1	d
Na8	Na	0.1646(12)	0.1520(3)	0.6029(5)	Uiso	0.0167(12)	4	1	d
O7	O	0.396(2)	0.3906(5)	0.6294(8)	Uiso	0.0171(19)	4	1	d
Na9	Na	0.1328(13)	0.4708(3)	0.6142(5)	Uiso	0.0221(12)	4	1	d
O8	O	0.173(2)	0.3156(5)	0.6500(8)	Uiso	0.020(2)	4	1	d
Na10	Na	0.4703(15)	0.1639(4)	0.7339(6)	Uiso	0.0202(9)	4	1	d
O9	O	0.261(2)	0.1586(5)	0.3236(8)	Uiso	0.018(2)	4	1	d
O10	O	0.186(2)	0.0046(5)	0.1539(8)	Uiso	0.024(2)	4	1	d
O11	O	0.146(2)	0.2579(5)	0.9076(8)	Uiso	0.022(2)	4	1	d
O12	O	-0.244(2)	0.3442(5)	0.3127(8)	Uiso	0.019(2)	4	1	d
Na11	Na	0.4741(12)	0.4839(3)	0.2368(5)	Uiso	0.0168(12)	4	1	d
O13	O	0.358(2)	0.2514(5)	0.5650(8)	Uiso	0.0148(18)	4	1	d
O14	O	0.096(2)	0.4152(5)	0.4106(8)	Uiso	0.0189(18)	4	1	d
Na12	Na	0.1306(13)	0.1766(3)	0.1065(5)	Uiso	0.0209(12)	4	1	d
O15	O	0.037(2)	0.4484(5)	0.8329(9)	Uiso	0.028(2)	4	1	d
O16	O	0.043(2)	0.1232(5)	0.8813(9)	Uiso	0.026(2)	4	1	d
O17	O	0.398(2)	0.0529(5)	0.6002(8)	Uiso	0.017(2)	4	1	d
O18	O	0.092(2)	0.0766(5)	0.3982(8)	Uiso	0.019(2)	4	1	d
C4	C	0.323(4)	0.3192(9)	0.6201(17)	Uiso	0.028(3)	4	1	d
C5	C	0.313(4)	0.3454(9)	0.1165(15)	Uiso	0.023(3)	4	1	d

C6	C	0.312(4)	0.0125(9)	0.1089(16)	Uiso	0.030(4)	4	1	d

**Refinement details**	
Phase	ε II
Chemical formula	Na_2CO_3
Crystal system	Monoclinic
Space group	Cc
a (P = 12.32(5) GPa)	8.526(3) Å
b (P = 12.32(5) GPa)	14.96930(10) Å
c (P = 12.32(5) GPa)	11.08720(10) Å
β	103.75(4)°
Cell volume (P = 12.32(5) GPa)	1374.5(5) Å³
Z	24
Density (P = 3 GPa)	3.07 g/cm³
Total number of reflections	1188
No. reflections I > 3σ(I)	943
R1	0.0607
R1 (all)	0.0748
wR	0.0674
wR (all)	0.0682
Number of parameters	145
GooF	3.63

S10: ε-II atomic positions and refinement details

**Step**	**P (GPa)**	**T (K)**	**a (Å)**	**b (Å)**	**c (Å)**	**B (°)**	**Volume (Å³)**	**Phase**
1	0(0.0009)	298	8.885(5)	5.2311(3)	5.999(5)	101.70(8)	273.0(3)	γ
2	0.50(5)	298	8.891(5)	5.2369(4)	5.996(6)	101.80(9)	273.3(3)	γ
3	1.31(5)	298	8.61(6)	5.142(6)	6.11(7)	101(1)	265(4)	γ
4	2.60(5)	298	8.802(13)	5.1946(7)	11.73(3)	102.5(2)	524(1)	ε
5	3.90(5)	298	8.739(9)	5.1979(6)	11.62(2)	103.16(17)	514(1)	ε
6	3.94(5)	341	8.707(13)	5.1950(6)	11.57(3)	103.2(2)	510(1)	ε
7	4.00(5)	381	8.713(14)	5.1924(8)	11.60(3)	102.8(2)	512(2)	ε
8	3.75(5)	469	8.767(10)	5.1780(6)	11.66(2)	102.76(18)	516(1)	ε
12	3.75(5)	531	8.716(11)	5.1585(6)	11.63(2)	102.8(2)	510(1)	ε
13	4.00(5)	595	8.783(4)	5.1769(3)	11.714(8)	102.49(7)	520.3(4)	ε
14	4.25(5)	641	8.820(3)	5.1989(2)	11.775(6)	102.35(5)	527.4(3)	ε
15	4.50(5)	690	8.776(17)	5.1688(13)	11.78(3)	102.1(3)	522(2)	ε

16	4.87(5)	308	8.713(17)	5.1683(7)	11.51(3)	103.7(3)	504(2)	ε
17	6.72(5)	303	8.622(14)	5.1105(7)	11.30(3)	105.3(2)	480(1)	ε
18	7.00(5)	555	8.54(2)	5.1195(19)	11.26(4)	105.0(4)	476(2)	ε
19	8.00(5)	665	8.68(4)	5.143(5)	11.37(10)	103.6(8)	494(5)	ε
20	9.00(5)	318	8.48(4)	5.080(2)	11.15(9)	105.0(8)	464(4)	ε

S11: Unit cell and volume variation with pressure and temperature in Run3. Esds in parentheses.

Step	P (GPa)	T K	a (Å)	b (Å)	c (Å)	B (°)	Volume (Å³)	q1	q2	Phase
1	0.07	296	8.9041(10)	5.2389(3)	6.02(1)	101.35(5)	275.3(4)	0.171(2)	0.327(3)	γ
2	0.07	296	8.8995(8)	5.2366(2)	6.030(6)	101.46(3)	275.4(3)	0.1804(7)	0.317(1)	γ
3	0.26	296	8.8920(9)	5.2330(3)	6.023(8)	101.52(4)	274.6(4)	0.1804(8)	0.316(1)	γ
4	0.3	312	8.8855(13)	5.2290(3)	6.023(8)	101.57(5)	274.1(4)	0.1802(6)	0.3164(3)	γ
5	0.28	337	8.8799(11)	5.2296(2)	6.017(7)	101.57(4)	273.8(3)	0.1800(6)	0.317(1)	γ
6	0.68	376	8.871(3)	5.2257(6)	6.017(16)	101.65(10)	273.2(7)	0.174(2)	0.3257(9)	γ
7	0.83	424	8.8737(17)	5.2274(4)	6.002(12)	101.63(7)	272.7(5)	0.1713(2)	0.329(3)	γ
8	1.22	474	8.866(2)	5.2250(4)	5.998(18)	101.67(10)	272.1(8)	0.174(2)	0.327(3)	γ
9	1.79	525	8.8430(16)	5.2125(6)	5.967(12)	101.91(7)	269.1(5)	0.174(2)	0.329(2)	γ
10	2.44	575	8.8280(19)	5.2098(4)	5.944(12)	102.07(7)	267.3(5)	0.1802(7)	0.332(1)	γ
11	2.42	582	8.825(2)	5.2083(5)	5.949(16)	102.03(10)	267.5(7)	0.176(2)	0.332(2)	γ
12	2.18	585	8.8389(14)	5.2133(5)	5.954(12)	102.05(6)	268.3(5)	0.175(2)	0.332(2)	γ
13	2.21	632	8.8457(12)	5.2195(4)	5.945(8)	101.94(5)	268.5(4)	0.176(2)	0.332(1)	γ
14	1.73	673	8.8623(14)	5.2179(5)	6.013(11)	101.48(6)	272.5(5)	0.177(2)	0.325(3)	γ
15	1.83	726	8.9002(16)	5.2365(4)	6.043(10)	101.13(7)	276.3(4)	0.1762(6)	0.3161(13)	γ
16	1.47	775	8.905(7)	5.2329(7)	6.06(3)	101.0(2)	277(1)	0.1730(10)	0.313(2)	γ
17	1.51	780	8.9010(19)	5.2330(5)	6.086(11)	100.78(7)	278.5(5)	0.1704(9)	0.311(2)	γ
18	1.51	831	8.880(6)	5.2164(14)	6.11(3)	100.5(2)	278(1)	0.1649(8)	0.305(2)	γ
19	1.56	883	8.9167(17)	5.2316(5)	6.133(9)	100.22(6)	281.5(4)	0.1570(10)	0.302(2)	γ
20	0.32	553	8.9370(17)	5.2506(3)	6.099(8)	100.72(6)	281.2(4)	0.1706(9)	0.311(2)	γ
21	0.58	691	8.9266(19)	5.2383(4)	6.133(9)	100.34(7)	282.1(4)	0.1668(8)	0.3042(18)	γ
22	0.66	793	8.956(2)	5.2501(4)	6.178(8)	99.87(6)	286.2(4)	0.1512(7)	0.2908(15)	γ
23	0.81	887	8.9802(15)	5.2412(3)	6.236(7)	98.49(5)	290.3(3)			β
24	0.84	901	8.989(2)	5.2378(4)	6.252(10)	97.90(6)	291.5(5)			β
25	0.74	948	9.001(5)	5.2199(6)	6.371(12)	95.66(11)	297.9(6)			β
26	0.92	1011	5.2088(7)		6.441(10)		151.4(2)			α
27	0.801	1020	5.2023(6)		6.444(10)		151.0(2)			α

S12: Unit cell and volume variation with pressure and temperature in Run4. Esds in parentheses.
